# Knockout of *Bmal1* in dopaminergic neurons induces ADHD-like symptoms via hyperactive dopamine signaling in male mice

**DOI:** 10.1186/s12993-025-00287-w

**Published:** 2025-07-11

**Authors:** Yichun Zhang, Xin Li, Yong Liu, Xiangyu Li, Dengfeng Liu, Qingyun Han, Xiran Liu, Xuyi Wang, Jia-Da Li, Suixin Deng

**Affiliations:** 1 Furong Laboratory, Center for Medical Genetics, School of Life Sciences, Central South University, Changsha, Hunan P. R. China; 2 Mental Health Institute, National Center of Mental Disorder, Second Xiangya Hospital of Central South University, Changsha, Hunan P. R. China; 3 MOE Key Laboratory of Rare Pediatric Diseases, Changsha, Hunan P. R. China; 4 Hunan Key Laboratory of Animal Models for Human Diseases, Changsha, Hunan P. R. China; 5 Hunan Key Laboratory of Medical Genetics, Changsha, Hunan P. R. China; 6 Hunan International Scientific and Technological Cooperation Base of Animal Models for Human Diseases, Changsha, Hunan P. R. China

**Keywords:** Circadian genes, BMAL1, ADHD, Dopamine, Dorsal striatum

## Abstract

**Background:**

The central circadian clock coordinates daily oscillations in physiology, metabolism and behavior. Disruptions to core circadian clock genes not only perturb sleep-wake rhythms but also contribute to psychiatric disorders. While dopaminergic dysfunction is strongly associated with mental illnesses, the mechanistic connection between circadian clock genes and dopamine signaling remains elusive. In the current study, we directly examine the role of the core circadian gene *Bmal1* in dopamine neurons, investigating its effects on behavioral outcomes and dopamine signaling.

**Results:**

*Bmal1* conditional knockout (cKO) mice specific to dopamine neuron were generated by crossing *Bmal1*-flox strain with the *Dat*-Cre strain, with knockout efficiency validated through immunofluorescence. BMAL1 deficiency in dopaminergic neurons induces attention-deficit hyperactivity disorder (ADHD)-like phenotypes, including hyperactivity, impairments in attention and working memory. Dopamine sensor detection revealed increased dopamine release in *Bmal1*-cKO mice. Additionally, electrophysiological recording showed that striatal neurons in *Bmal1* knockout mice exhibited increased neuronal excitability. Amphetamine and dopamine D1 receptor antagonist SCH23390 treatment attenuated the hyperactivity behavior in cKO mice.

**Conclusions:**

This study finds that BMAL1 ablation in dopaminergic neurons induces ADHD-like phenotypes in male mice, identifying hyperactive dopamine signaling as a potential mediator of these phenotypes. It unveils a novel role for BMAL1 in regulating dopamine signaling and provide insights into circadian gene-driven psychiatric pathophysiology.

**Supplementary Information:**

The online version contains supplementary material available at 10.1186/s12993-025-00287-w.

## Introduction

A circadian clock is an approximately 24-h daily period endogenous oscillator that regulates physiological, metabolic and behavioral processes across tissues and cells [1, 2]. At the molecular level, the circadian clock operates primarily through a transcriptional-translational feedback loop regulated by a core set of circadian genes. Disruptions of this molecular machinery not only affects the circadian rhythm (e.g., sleep-wake cycle) but also increases susceptibility to psychiatric disorders, such as depression, anxiety and bipolar disorder [3, 4]. Despite these associations, the causal mechanisms linking circadian gene dysfunction to mental illnesses remain poorly defined.

In mammals, the circadian network is centered on two transcription factors: *CLOCK* (circadian locomotor output cycles kaput) and *BMAL1* (brain and muscle aryl hydrocarbon receptor nuclear translocator-like 1). CLOCK and BMAL1 form a heterodimer and bind to E-box elements (CACGTG) located in the promoters of genes including *PERIOD* (*PER1-3*) and *Cryptochrome* (*CRY1/2*) [5, 6]. Subsequently, PER and CRY proteins form a complex that repress the activity of CLOCK/BMAL1 heterodimer [7]. Furthermore, Rev-erbs and retinoic acid receptor-related orphan receptor (Ror) proteins form an auxiliary regulatory loop by repressing or activating Bmal1 transcription, respectively [7]. Among these components, Bmal1 acts as a key generator of the circadian clock, and global knockout of BMAL1 in rodents or monkeys leads to abnormal rhythmic behaviors and psychiatric disorders [8, 9].

The dopaminergic system regulates motor control, motivation, reward and cognitive functions [10]. Notably, dopamine synthesis and secretion follow a diurnal cycle synchronized with the circadian rhythm of clock genes and proteins. Emerging evidence suggests that mutations or ablation of circadian genes, such as *Clock*,* Cry1* and *Rev-erbα*, dysregulate dopaminergic system functions, including enhanced dopamine release [11] and hyperactive dopamine D1 receptor signaling [12]. These alterations may contribute to the risk of psychiatric disorders, such as mood disorders, substance use disorders, and schizophrenia, which are often associated with circadian and sleep disturbances affecting cognition, motivation, and impulsivity [13]. Further studies demonstrate circadian genes modulate the dopamine homeostasis through catabolic pathways. For example, monoamine oxidase A (MaoA), an enzyme involved in dopamine catabolism, is regulated by clock components Bmal1, Npas2, and Per2. Therefore, Bmal1 could regulate the mood states via a direct modulation of dopaminergic functions.

To explore the role of BMAL1 specifically in dopaminergic neurons and its implications for psychiatric disorders, we generated mice with conditional knockout of *Bmal1* to investigate the underlying mechanisms. We show that BMAL1 ablation in dopaminergic neurons induces ADHD-like phenotypes and identify that hyperactive dopamine signaling as a potential mediator of these phenotypes.

## Results

### *Bmal1* ablation in dopaminergic neurons results no alteration in circadian period

**Fig. 1:**
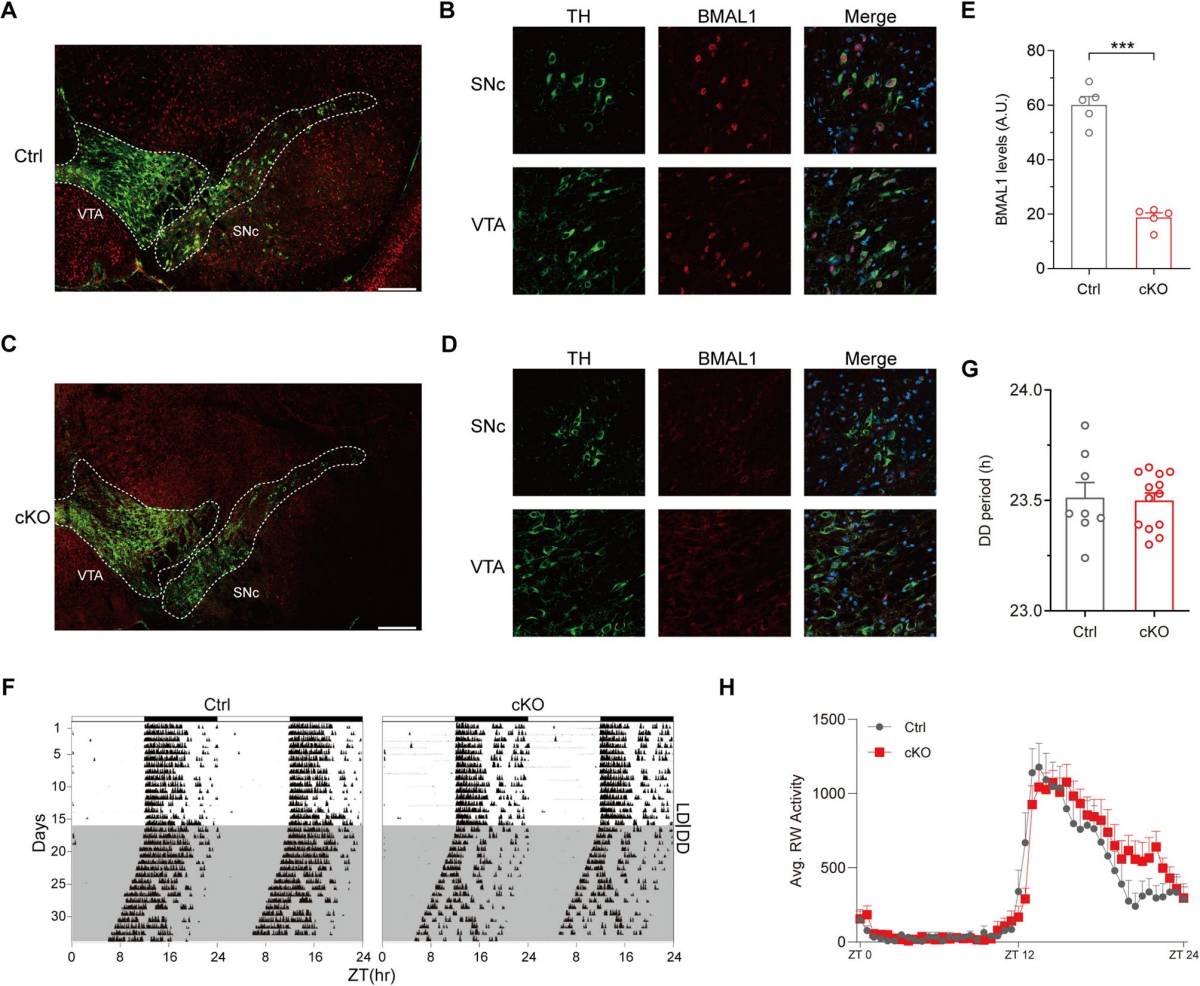
Conditional knockout of *Bmal1* in the midbrain and the circadian rhythm in cKO mice. (**A**) Representative immunofluorescence image of BMAL1 expression in SNc and VTA brain regions of control (Ctrl) mice. Green represents TH protein, and red represents BMAL1 protein. Scale bar: 300 μm. (**B**) High magnification images showing BMAL1 expression in TH-positive neurons in the SNc and VTA of Ctrl mice. Scale bar: 10 μm. (**C**) Representative immunofluorescence image of BMAL1 expression in SNc and VTA brain regions of cKO mice. Green represents TH protein, and red represents BMAL1 protein. Scale bar: 300 μm. (**D**) High magnification images showing BMAL1 expression in TH-positive neurons in the SNc and VTA of cKO mice. Scale bar: 10 μm. (**E**) The quantification of fluorescence intensity of BMAL1 was analyzed by ImageJ. ***P < 0.001, unpaired Student’s t test. *n* = 5 per genotype. (**F**) Representative double-plotted actograms of Ctrl and cKO mice. (**G**) The free-running periods of Ctrl (*n* = 8) and cKO (*n* = 13) mice; *P* > 0.05, unpaired Student’s t test. (**H**) Average wheel-running (RW) activity of Ctrl (*n* = 8) and cKO (*n* = 13) mice recorded for 5 days plotted in 30-min bins. Data are presented as mean ± SEM

To generate *Bmal1* conditional knockout mice (*Bmal1*-cKO; *Dat*-Cre^+^/*Bmal1*-flox^+/+^) and control mice (*Dat*-Cre^−^/*Bmal1*-flox^+/+^), we crossed the *Bmal1*-flox carrying strain with the *Dat*-Cre carrying strain. Then, we performed immunofluorescence analysis to validate the knockout efficiency of BMAL1 in dopamine neurons. Tyrosine hydroxylase (TH), a key enzyme in dopamine synthesis, is expressed in nearly all dopamine neurons. Therefore, we identified dopamine neurons located at substantia nigra pars compacta (SNc) and ventral tegmental area (VTA) via TH immunofluorescence (Fig. 1A-D). In control mice, TH-positive dopamine neurons co-expressed BMAL1 (Fig. 1A and B). Whereas in cKO mice, TH-positive dopamine neurons lacked BMAL1 expression (Fig. 1C and D). Quantitative analysis revealed a significant decrease in BMAL1 fluorescence intensity in cKO mice compared to controls (60.06 ± 3.15 versus 18.8 ± 1.68 A.U., *p* < 0.001, unpaired Student’s t test; Fig. 1E). Notably, no reduction in BMAL1 expression was observed in brain regions beyond the SNc and VTA, including the prefrontal cortex (PFC) and dorsal striatum, where *Dat*-Cre-mediated recombination is not anticipated (Supplementary Fig. 1A and B). These results confirm the specific and efficient knockout of BMAL1 in TH-positive neurons in *Bmal1*-cKO mice.

As a core circadian gene, Bmal1 is essential for normal circadian behavior and its expression varies within a daily cycle. However, the impact of BMAL1 on circadian rhythm in dopamine neurons is still unknown. Here, we monitored the wheel-running activity of *Bmal1*-cKO mice and their littermate controls. Animals were housed individually in cages equipped with running wheels. Wheel-running activities were continuously recorded for 2–3 weeks under a 12-hour light/dark (LD) cycle, followed by a switch to constant darkness (DD) for 3–4 weeks. We observed no significant difference in the free-running periods under DD between control and cKO mice (23.51 ± 0.07 versus 23.5 ± 0.03 h, *p* = 0.183, unpaired Student’s t test), indicating that BMAL1 in dopamine neurons has a negligible impact on circadian period (Fig. 1F-H). We also analyzed the expression of core circadian genes *Clock*, *Per2*, and *Cry1* in midbrain tissue and found no significant differences between cKO and control mice (Supplementary Fig. 1C), suggesting that *Bmal1* deletion in dopaminergic neurons does not broadly disrupt the expression of other core clock genes.

### Knockout of *Bmal1* in dopamine neurons causes hyperactivity and cognitive deficits

Regardless of the negligible impact on circadian period in *Bmal1*-cKO mice, we next investigated the behavioral consequences of selective *Bmal1* ablation in dopaminergic neurons using other behavioral tests to assess locomotor activity, cognitive function and social behavior [14]. Previous report indicates that BMAL1 expression in ventral midbrain is elevated at night and reduced during the daytime. Therefore, we performed a series of behavioral tests on *Bmal1*-cKO mice and their littermate controls during two different zeitgeber time (ZT) periods: at the end of the light phase (ZT09-12) and dark phase (ZT21-24).

**Fig. 2:**
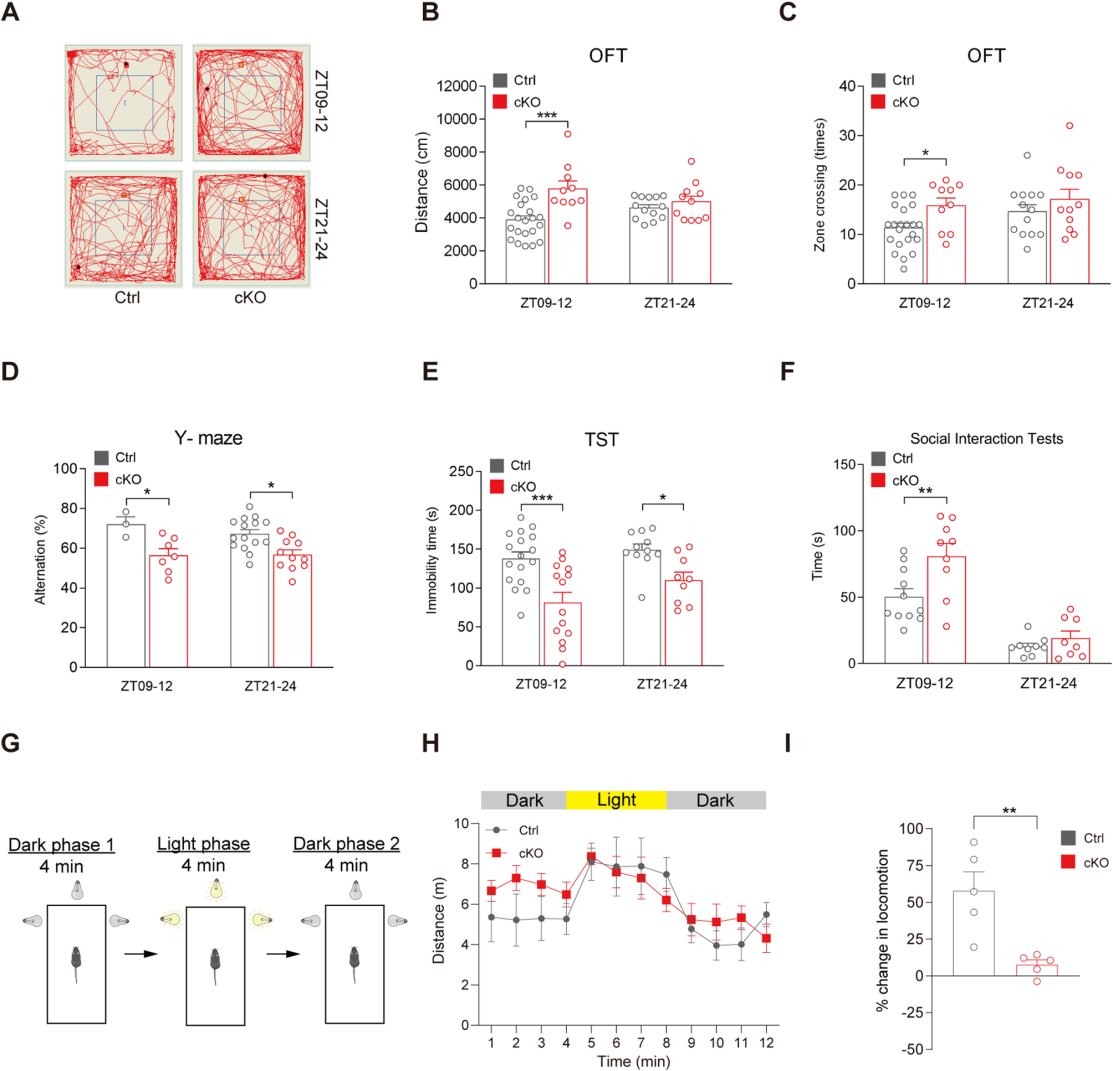
Behavioral consequences of selective *Bmal1* ablation in dopaminergic neurons. (**A**) Representative movement traces of animals in the OFT. (**B**) cKO mice travelled significantly longer distances than Ctrl mice in the OFT at ZT09-12; ***P < 0.001, two-way ANOVA test. ZT09-12, *n* = 22 versus 10 mice; ZT21-24, *n* = 13 versus 11 mice. (**C**) The number of zone crossings in the OFT was significantly higher in cKO mice at ZT09-12; *P < 0.05, two-way ANOVA test. ZT09-12, *n* = 22 versus 10 mice; ZT21-24, *n* = 13 versus 11 mice. (**D**) Spontaneous alternation in the Y-maze test was significantly reduced in cKO mice compared to Ctrl mice; *P < 0.05, two-way ANOVA test. ZT09-12, *n* = 3 versus 7 mice; ZT21-24, *n* = 15 versus 11 mice. (**E**) Immobility time in TST was significantly shorter in cKO mice; *P < 0.05, ***P < 0.001, two-way ANOVA test. ZT09-12, *n* = 17 versus 14 mice; ZT21-24, *n* = 11 versus 9 mice. (**F**) Social interaction time in the reciprocal social interaction test was significantly longer in cKO mice at ZT09-12; **P < 0.01, two-way ANOVA test. ZT09-12, *n* = 12 versus 9 mice; ZT21-24, *n* = 9 versus 8 mice. (**G**) The schematic diagram of bright-light stimulation test. (**H**) Distance traveled in bright-light stimulation test. cKO group mice showed less response in ambulation in response to light stimulation. *n* = 5 versus 5 mice. (**I**) The percentage of change in locomotion. *n* = 5 versus 5 mice, **P < 0.01, unpaired Student’s t test. Data are presented as mean ± SEM

Firstly, we used an open field test (OFT) to measure spontaneous locomotor activity. During the ZT09-12 period, cKO mice exhibited significantly greater movement, as evidenced by longer distances traveled and increased zone crossings, compared to control mice (Distance traveled: 3880.45 ± 239.77 versus 5771.14 ± 478.66 cm, *p* < 0.001, two-way ANOVA test; Zone crossings: 11.56 ± 0.92 versus 15.8 ± 1.53 times, *p* = 0.030, two-way ANOVA test). Meanwhile, no obvious differences were observed (Distance traveled: 4619.22 ± 184.30 versus 4988.75 ± 347.61 cm, *p* = 0.853, two-way ANOVA test; Zone crossings: 14.62 ± 1.42 versus 17.09 ± 2.10 times, *p* = 0.327, two-way ANOVA test) when the test was performed at ZT21-24 (Fig. 2A-C). These results indicate that *Bmal1* deficiency in dopaminergic neurons causes hyperactivity in a circadian time-dependent manner. In addition, Bmal1 global knockout mice are known to show deficits in habituation to novel environment in OFT [15]. To dissociate true hyperactivity from slowed habituation of Bmal1 cKO mice, we performed the OFT for 2 consecutive days at ZT09-12. Unlike Bmal1 global knockout mice, our result shows that *Bmal1*-cKO mice display normal habituation to the novel environment (Supplementary Fig. 2A-D).

Previous studies have shown that global knockout of *BMAL1* impair short-term memory and lead to despair-like phenotypes [16, 17]. Therefore, we conducted a Y-maze assay which evaluates attention, learning and memory. In this assay, cKO mice showed significantly reduced correct rate than control mice during both ZT09-12 (72.04 ± 3.71 versus 56.52 ± 3.28 alternation%, *p* = 0.041, two-way ANOVA test) and ZT21-24 (67.26 ± 2.20 versus 56.84 ± 2.36 alternation%, *p* = 0.013, two-way ANOVA test) periods (Fig. 2D), suggesting deficits in working memory. We then analyzed despair-like behaviors by using the tail suspension test (TST). In this test, cKO mice exhibited significantly less immobility time than control mice during both time periods (ZT09-12: 138.53 ± 8.32 versus 81.71 ± 13.03 s, *p* < 0.001; ZT21-24: 149.36 ± 7.35 versus 110.44 ± 10.34 s, *p* = 0.006, two-way ANOVA test), indicating reduced despair-like behavior (Fig. 2E).

To evaluate social behaviors, we also performed reciprocal social interaction test in cKO mice and their controls. During the ZT09-12 period, cKO mice spent significantly more time engaging in social interactions compared to control mice (50.45 ± 6.12 versus 81.11 ± 2.32 s, *p* = 0.007, two-way ANOVA test). However, no obvious difference was observed during the ZT21-24 period (12.96 ± 2.32 versus 19.25 ± 5.27 s, *p* = 0.909, two-way ANOVA test; Fig. 2F).

Recent studies reported a bright-light stimulation test to assess the attention to environmental stimuli of cKO mice. The appearance of bright light simulates the sudden emergence of a natural predator, which can automatically induce exogenous attention [18, 19]. We then preformed the bright-light stimulation test at ZT09-12 to test the attention to environmental stimuli of cKO mice (Fig. 2G). In the test, cKO mice showed increased locomotion in the first dark phase compared to control mice. However, cKO mice response less to the light stimulation (Fig. 2H, the change of distance traveled from 4 to 5 min). Together with the locomotion change between light phase and dark phase 1(58 ± 12.67 versus 7.58 ± 3.26%, *p* = 0.049, unpaired Student’s t test), these results indicate that cKO mice exhibit impaired attention (Fig. 2I).

Taken together, these findings reveal several behavioral abnormalities in *Bmal1*-cKO mice, including hyperactivity, impaired working memory, attention deficits, altered despair-like behavior, and abnormal social behavior. Notably, the hyperactivity, as well as impaired attention and working memory, closely resemble the hallmark symptoms of ADHD, a highly heritable neurodevelopmental disorder.

### Increase in dopamine release in *Bmal1* conditional knockout mice

Dopamine released by mesencephalic dopamine neurons plays an important role in regulating motor control, motivation, reward and cognitive functions. The ADHD-like behaviors observed in *Bmal1*-cKO mice in our study may therefore result from dysregulated of dopamine release. To test this hypothesis, we investigated both the homeostasis and release dynamics of dopamine.

**Fig. 3:**
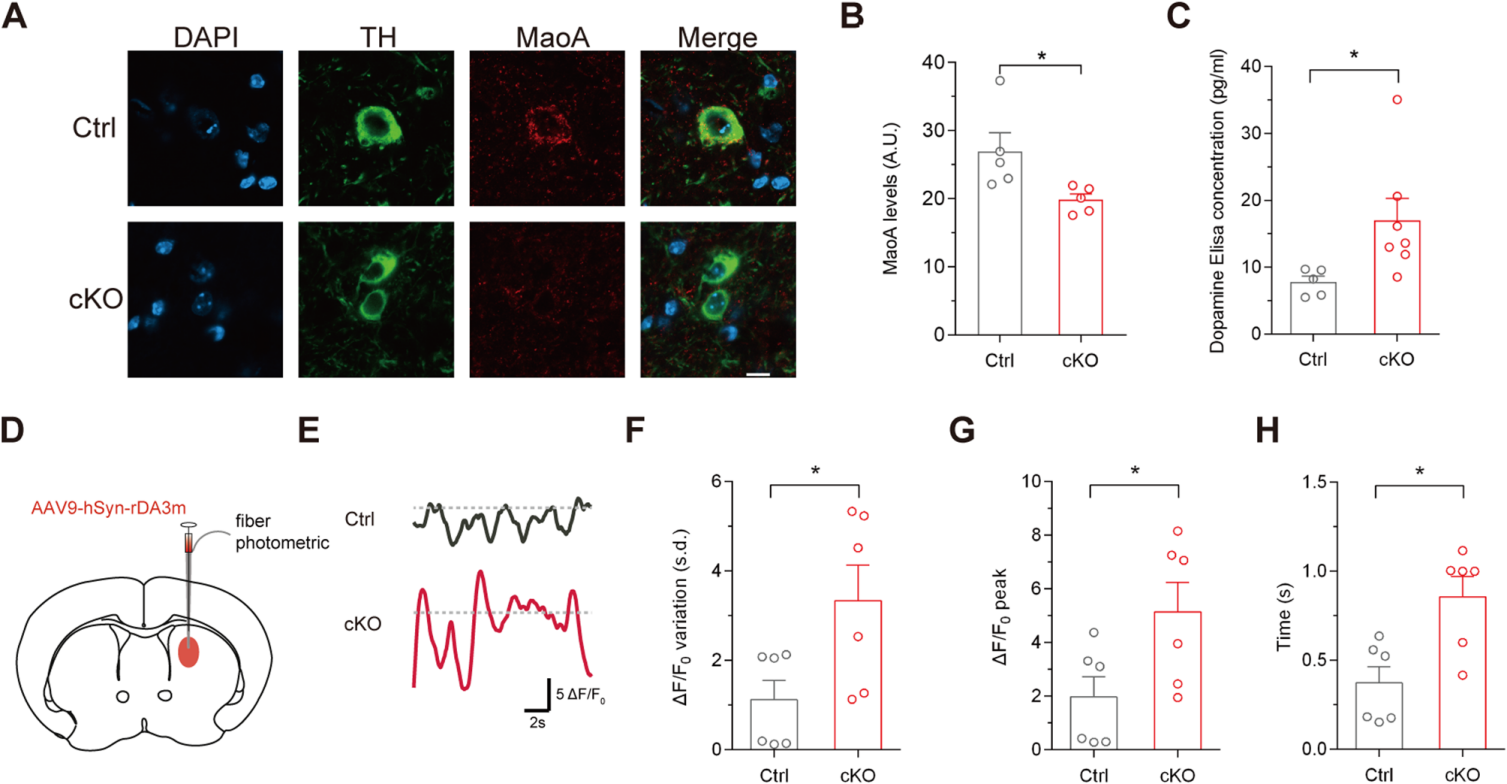
Dopamine homeostasis and release dynamics in *Bmal1*-cKO mice. (**A**) Representative immunofluorescence images of MaoA expression in TH-positive neurons in the VTA of Ctrl and cKO mice. Scale bar: 10 μm. (**B**) The quantification of fluorescence intensity of MaoA was analyzed by ImageJ. *P < 0.05, unpaired Student’s t test. *n* = 5 per genotype. (**C**) ELISA test for the contents of dopamine in the downstream nuclei (nucleus accumbens) of dopaminergic neurons in Ctrl (*n* = 5) and cKO (*n* = 7) mice; *P < 0.05, unpaired Student’s t test. (**D**) Schematic diagram of the experimental setup. The fiber photometric canula was inserted into the right dorsal striatum. (**E**) Example traces of rDA3m fluorescence. Dotted lines indicate the positive standard deviation value (s.d., threshold for high-level dopamine release bouts) of ΔF/F_0_. (**F-H**) Quantification of fluorescence variation measured as (**F**) the s.d., (**G**) the peak amplitude, and (**H**) the peak duration of ΔF/F_0_ of rDA3m fluorescence. Each data point represents one 30-second trace analyzed. *P < 0.05, unpaired Student’s t test. Data are presented as mean ± SEM

At the cellular level, the promoter of monoamine oxidase A (MaoA), an enzyme involved in dopamine catabolism, is regulated by the circadian clock components including BMAL1 [20]. Thus, we examined the expression level of MaoA in the midbrain of cKO mice and their littermate controls using immunofluorescence analysis. We observed a significant decrease of MaoA expression in TH-positive neurons (Fig. 3A), indicating that BMAL1 positively regulates MaoA expression, consistent with previous cellular experiments. The fluorescence intensity of MaoA has been calculated (26.94 ± 2.74 versus 19.86 ± 0.86 A.U., *p* = 0.039, unpaired Student’s t test; Fig. 3B). Furthermore, ELISA analysis revealed a significant increase of dopamine level (7.78 ± 0.89 versus 16.99 ± 3.33 pg/ml, *p* = 0.047, unpaired Student’s t test) in the downstream nuclei of midbrain in cKO mice (Fig. 3C). These results suggest that the ablation of BMAL1 in dopaminergic neurons downregulates MaoA expression, leading to dopamine accumulation, which may subsequently enhance dopamine release.

To directly measure the alterations in dopamine release, we used a well-established dopamine sensor, rDA3m [21, 22], to monitor dopamine dynamics in brain nuclei downstream of midbrain dopaminergic neurons. Dopamine neurons in the SNc project to the dorsal striatum, forming the nigrostriatal pathway, which controls movements and motivated behaviors. Using fiber photometry, we monitored dopamine dynamics in the dorsal striatum of control and *Bmal1*-cKO mice in OFT (Fig. 3D). During the resting state of OFT, dopamine fluctuations, measured as variations (1.12 ± 0.43 versus 3.34 ± 0.79 s.d., *p* = 0.034, unpaired Student’s t test) in rDA3m fluorescence (ΔF/F_0_) [23], were markedly increased in cKO mice (Fig. 3E and F). Indicating hyperactive dopamine dynamics due to selective ablation of *Bmal1* in dopaminergic neurons. Additionally, by analyzing the positive standard deviation (s.d.) of ΔF/F_0_ as a threshold to quantify dopamine fluctuation bouts, we found that cKO mice exhibited larger peak amplitudes (1.97 ± 0.75 versus 5.15 ± 1.10%, *p* = 0.038, unpaired Student’s t test) and longer durations of dopamine release events (0.37 ± 0.09 versus 0.86 ± 0.11 s, *p* = 0.008, unpaired Student’s t test) compared to controls (Fig. 3G and H).

Together, these findings highlight the critical role of *Bmal1* in maintaining dopaminergic homeostasis and regulating dopamine release dynamics, suggesting potential implications for neuropsychiatric disorders associated with dysregulated dopamine signaling.

### Enhanced neuronal excitability of striatal MSNs in *Bmal1* conditional knockout mice

**Fig. 4:**
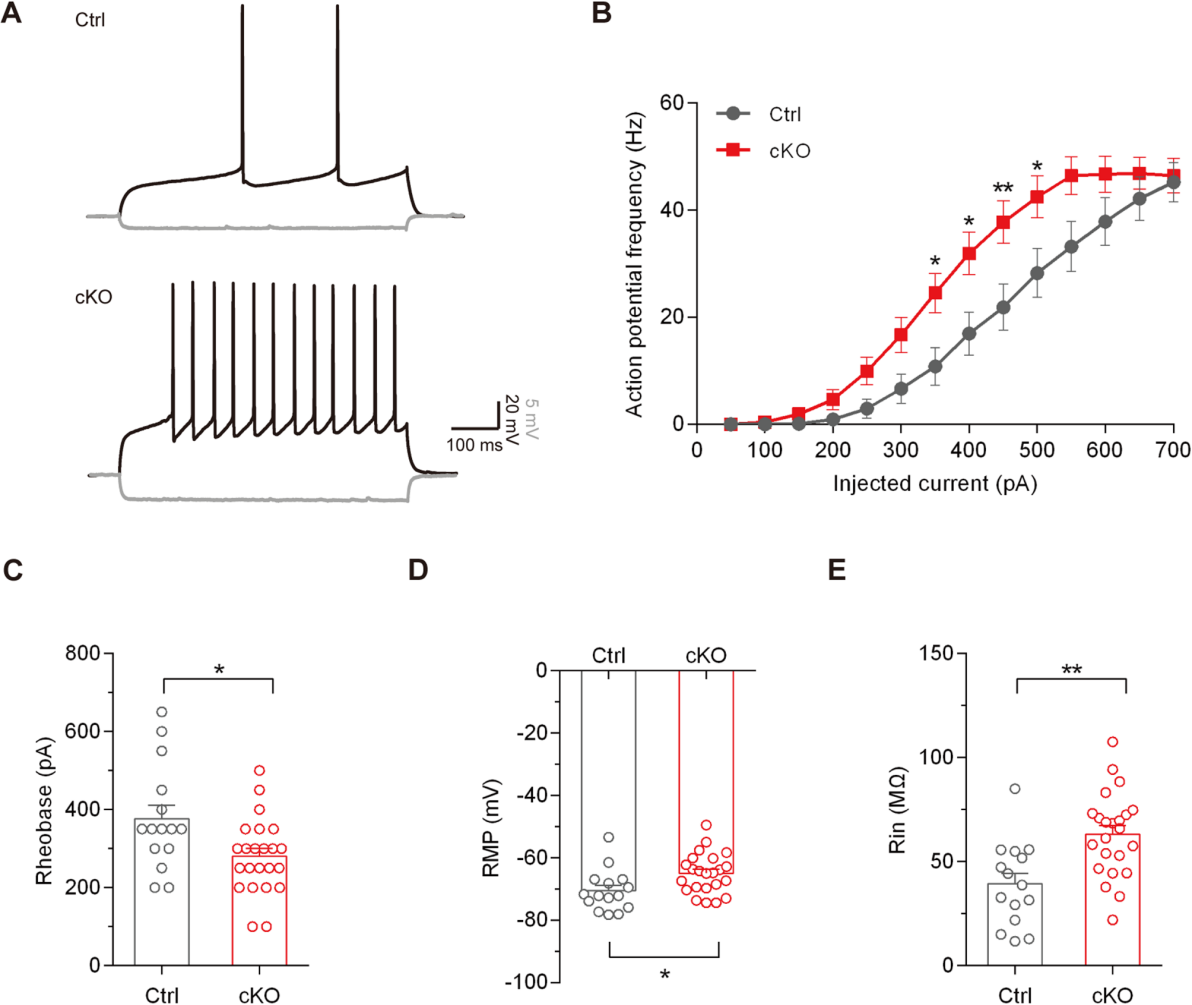
Striatal MSNs exhibit increased neuronal excitability in *Bmal1*-cKO mice. (**A**) Representative APs of dorsal striatum neurons induced by current injections of 350 pA (black) or -50 pA (grey) in Ctrl and cKO mice. The recording was performed at ZT09-12 timepoint. (**B**) The AP frequencies in response to a series of current pulses injections in dorsal striatum neurons from two groups. *n* = 15 neurons from 5 Ctrl mice versus 23 neurons from 6 cKO mice; **P < 0.01, *P < 0.05, repeated-measure ANOVA test. (**C**) The rheobase of dorsal striatum neurons from two groups. *P < 0.05, unpaired Student’s t test. (**D**) The resting membrane potential (RMP) of dorsal striatum neurons from two groups. *P < 0.05, unpaired Student’s t test. (**E**) The input resistance (Rin) of dorsal striatum neurons from two groups. **P < 0.01, unpaired Student’s t test. Data are presented as mean ± SEM

Considering enhanced dopamine release is likely to increase dopamine signaling, which would modulate the neuronal activities of dorsal striatum cells, we performed the whole-cell patch-clamp recordings to evaluate the excitability of medium spiny neurons (MSNs) in the dorsal striatum. Indeed, MSNs from cKO mice exhibited significantly greater excitability compared to those from control mice, as evidenced by higher action potential (AP) frequency in response to a series of current pulses injections (Fig. 4A and B), decreased rheobase current (376.67 ± 34.8 versus 280.45 ± 20.06 pA, *p* = 0.014, unpaired Student’s t test; Fig. 4C), more depolarized resting membrane potential (RMP) (-70.55 ± 1.72 versus − 64.98 ± 1.34 mV, *p* = 0.015, unpaired Student’s t test; Fig. 4D), and increased input resistance (Rin) of neurons (39.28 ± 5.19 versus 63.07 ± 4.2 MΩ, *p* = 0.001, unpaired Student’s t test; Fig. 4E). In addition, we performed whole-cell patch-clamp recordings to evaluate the excitability of pyramidal cells in the PFC, and no significant differences in neuron excitability were observed between two groups (Supplementary Fig. 3A-E). Collectively, our data indicate that the nigrostriatal pathway is hyperactive in cKO mice, suggesting that dysregulated dopamine release and signaling contribute to altered neuronal function in dorsal striatum.

### Pharmacological rescue of hyperactivity behavior via modulation of dopamine signaling

**Fig. 5:**
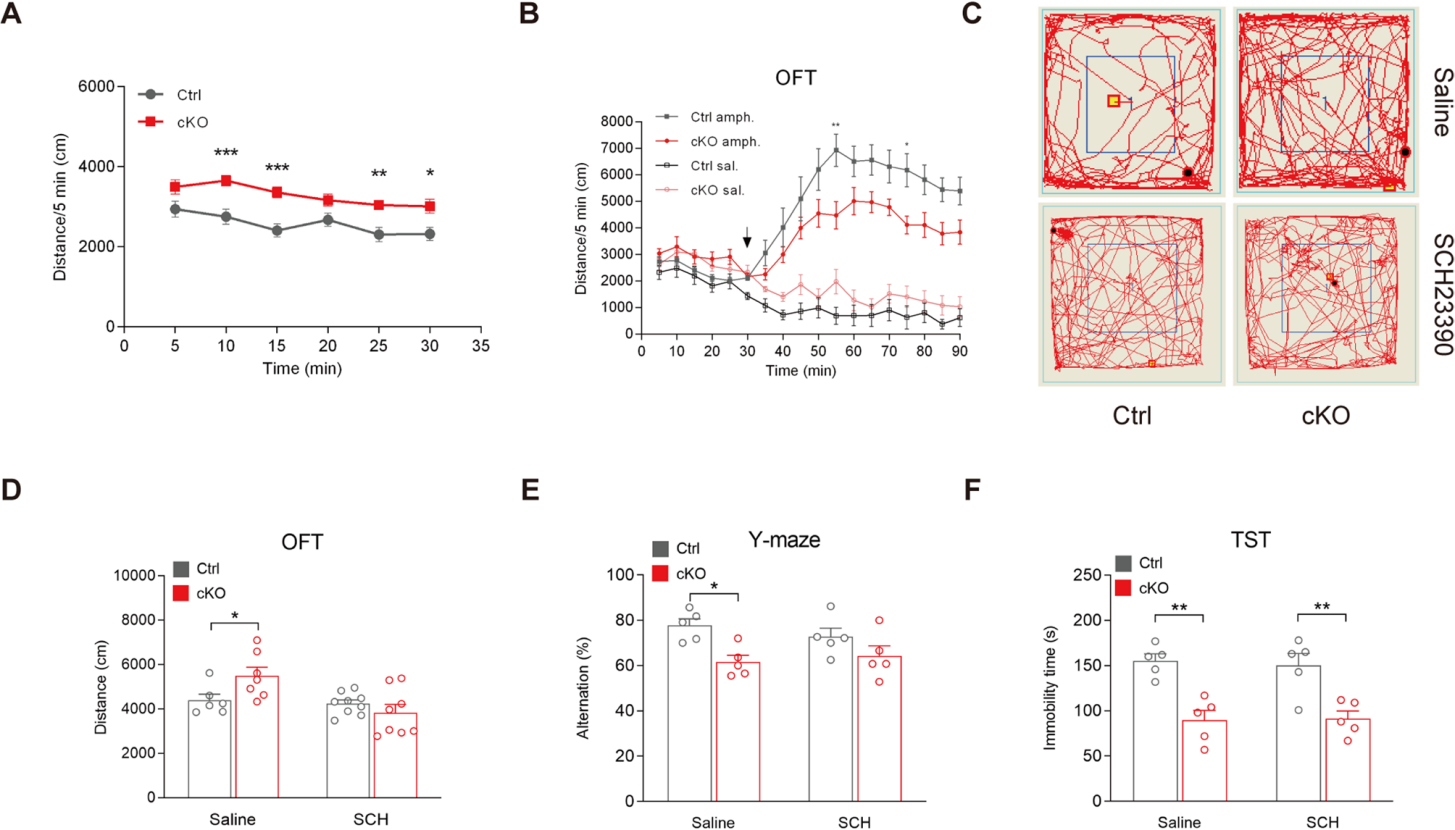
Amphetamine and D1 receptor antagonist attenuate the hyperactivity behavior in cKO mice. (**A**) cKO mice traveled significantly longer distances than Ctrl mice during a 30-minute OFT; ***P < 0.001, **P < 0.01, *P < 0.05, repeated-measure ANOVA test. *n* = 16 mice per genotype. (**B**) After 30 min of basal activity, amphetamine (0.2 mg/kg) or saline was injected i.p. (indicated by the arrow) and locomotor activity was monitored for 60 min. cKO mice travelled significantly shorter distances than Ctrl mice after amphetamine injection; **P < 0.01, *P < 0.05, repeated-measure ANOVA test. Saline: *n* = 5 Ctrl versus 6 cKO mice; Amphetamine: *n* = 10 Ctrl versus 11 cKO mice. (**C**) Representative movement traces of animals in the OFT after saline or SCH23390 treatment. (**D**) Saline or dopamine D1 receptor antagonist SCH23390 (15 µg/kg) were injected i.p., and behaviors were measured 30 min post-injection. SCH23390 normalized the hyperactivity in the OFT. Saline, *n* = 6 Ctrl versus 7 cKO mice; SCH, *n* = 9 Ctrl versus 8 cKO mice. *P < 0.05, two-way ANOVA test. Data are presented as mean ± SEM. (**E**) SCH23390 cannot normalized the reduced alteration in the Y-maze. *n* = 5 mice per genotype and treatment. *P < 0.05, two-way ANOVA test. (**F**) SCH23390 cannot normalized the anti-despair behavior in TST. *n* = 5 mice per genotype and treatment. **P < 0.01, two-way ANOVA test. Data are presented as mean ± SEM

To further investigate the long-term hyperactive behavioral phenotype of *Bmal1*-cKO mice and test the rescue effects of pharmacological interventions on dopamine signaling, we conducted a 30-minute OFT on both control and cKO mice. During this test, we recorded the distance traveled by the mice every 5 min. The results revealed that cKO mice traveled significantly greater distance in the OFT during each 5-minute interval compared to control mice (Fig. 5A).

Next, we challenged mice with amphetamine in a long-term OFT to explore the functional consequences of altered dopamine homeostasis. Amphetamine is commonly used as a treatment for ADHD [24] and is also a compound that elevates extracellular dopamine level [25]. Mice were acutely treated with intraperitoneal (i.p.) injections of either amphetamine (0.2 mg/kg) or saline 30 min after the onset of OFT. Before amphetamine administration, cKO mice exhibited significantly greater locomotor activity compared to control mice, consistent with our previous findings. However, following amphetamine injections, cKO mice traveled significantly shorter distances than control mice (Fig. 5B), indicating an alleviation of hyperactivity by amphetamine. The reduced responsiveness to amphetamine in cKO mice, coupled with their baseline hyperactivity, supports the notion of elevated dopamine levels under normal conditions, indicating lower resilience in behavior response to dopamine dynamic when *Bmal1* is lost in dopamine neurons.

Finally, we attempted to rescue the hyperactivity by reducing hyperactive dopamine signaling in cKO mice. We treated mice with the dopamine D1 receptor antagonist SCH23390 (15 µg/kg, i.p., a relatively low dose, which is expected to have minimal impact on behavior in wildtype mice) [26] and assessed their behavior 30 min later in the OFT. The results shown that SCH23390 normalized the hyperactivity of cKO mice in OFT (Saline: 4391.05 ± 270.63 versus 5496.51 ± 386.02 cm, *p* = 0.040; SCH: 4237.56 ± 163.49 versus 3829.13 ± 376.79 cm, *p* = 0.320, two-way ANOVA test; Fig. 5C and D). However, SCH23390 cannot rescue the abnormal behavior of cKO mice in Y-maze (Saline: 77.68 ± 2.97 versus 61.52 ± 2.98 alternation%, *p* = 0.040; SCH: 72.72 ± 3.83 versus 64.22 ± 4.51 alternation%, *p* = 0.530, two-way ANOVA test) and TST (Saline: 155.4 ± 7.64 versus 89.6 ± 10.96 s, *p* = 0.002; SCH: 150 ± 13.47 versus 91.4 ± 8.51 s, *p* = 0.005, two-way ANOVA test; Fig. 5E and F). These data indicate that enhanced dopamine signaling, particularly through D1 receptor-mediated pathways, play a critical role in the hyperactivity phenotype observed in *Bmal1*-cKO mice.

## Discussion

In this study, we demonstrated that selective ablation of *Bmal1* in dopaminergic neurons induces ADHD-like symptoms, including hyperactivity and cognitive deficits, without causing obvious disruption in circadian period in male mice. Our findings revealed that dopamine release is significantly increased in cKO mice. Furthermore, we identified a critical role for D1 receptor signaling in the dorsal striatum in mediating hyperactivity, as treatment with D1 receptor antagonist effectively normalized the hyperactive behavior in these mice. These results suggest a potential therapeutic strategy for alleviating ADHD-like core symptoms, such as hyperactivity, by modulating the dopaminergic system and targeting of *Bmal1*.

### Bmal1 ablation in dopamine neurons fails to alter circadian period

As a core gene of the circadian rhythm, *Bmal1* plays a critical role in maintaining normal circadian function, and its abnormal expression often leads to circadian rhythm disruptions. For instance, conventional *Bmal1* knockout mice exhibit completely disrupted circadian rhythms under constant darkness conditions [16]. Similarly, global *Bmal1* knockout in monkeys also results in reduced sleep and disrupted circadian rhythms [9]. Whereas, numerous studies have shown that the loss of *Bmal1* in different tissues or neuron types can heterogeneously affect circadian rhythms. Specifically, forebrain-specific *Bmal1* knockout mice exhibit circadian rhythm disruptions under both constant darkness and constant light conditions [27]. Mice with *Bmal1* specifically knocked out in the suprachiasmatic nucleus show an extended circadian period under constant darkness [28].

However, the absence of the *Bmal1* gene in certain neuron populations does not significantly affect the circadian rhythms. For instance, mice with *Bmal1* specifically knockout in corticotropin-releasing hormone neurons of the hypothalamic paraventricular nucleus maintain normal circadian rhythms under constant darkness [29]. Our results extend this understanding by showing that the specific knockout of *Bmal1* in dopaminergic neurons does not alter circadian period in mice under constant darkness, further highlight tissue-specific roles of *Bmal1* in physiological regulation.

### The role of the *Bmal1* gene in psychiatric disorders

The *BMAL1* gene is associated with many psychiatric disorders. Research indicates that BMAL1 knockout mice exhibit changes in motor-related behaviors. For instance, *Bmal1* conventional knockout mice show increased spontaneous activity in the OFT and impaired short-term memory in the novel object recognition test [30]. Another study found that the social abilities of *Bmal1* conventional knockout mice are significantly impaired, as evidenced by reduced social interaction in both reciprocal interaction and three-chamber tests, along with stereotyped grooming behaviors [14].

Moreover, specific deletion of the *Bmal1* gene in different brain regions results in diverse psychiatric manifestations in mice. One study reported that mice with *Bmal1* specifically knocked out in the striatum exhibit impaired motor coordination and an antidepressant phenotype [16]. In our study, we found that *Bmal1*-cKO mice, which lack BMAL1 expression specific in dopaminergic neurons, exhibit increased spontaneous movement, impaired attention and working memory, altered despair-like behavior, and abnormal social behavior. D1R antagonist reduced hyperactivity in the OFT but had no effect on TST performance, suggesting that the TST may reflect a separate behavioral component linked to despair, not just general hyperactivity. Furthermore, the prominent hyperactivity and cognitive deficits observed in cKO mice resemble key features of ADHD-like phenotype. Previous study has suggested a potential association between *Clock* gene mutation and ADHD susceptibility [31]. Given that BMAL1 and CLOCK are known binding partners and are believed to function together, they may jointly regulate dopamine level and thereby contribute to ADHD-related pathophysiology.

In addition, emerging evidence suggests that disruptions in circadian rhythm cause mental disorders primarily through dysregulation of circadian genes [8]. However, since no change in circadian period was observed in *Bmal1*-cKO mice, our results highlight a major causal role of circadian genes in mood disorders independent of their effects on circadian rhythm regulation.

### The role of circadian genes in dopaminergic functions and behavioral phenotypes

Circadian genes play a crucial role in regulating dopaminergic functions, which in turn influences behavioral phenotypes. The knockout or mutation of several circadian genes can lead to alterations in dopaminergic homeostasis mainly through the regulation of dopamine catabolism thus release. For instance, the transcription of *Mao* and *Dbh* (dopamine β-hydroxylase), two key genes involved in dopamine catabolism, is activated by the CLOCK: BMAL1 heterodimer but suppressed by *Per1b* [11]. Our results indicate that BMAL1 deficiency in the midbrain leads to downregulation of MaoA expression, resulting in dopamine accumulation. This finding was supported by fiber photometry data, which showed increased dopamine release in cKO mice. In addition, a previous study has shown that knockdown of CLOCK in the VTA results in behavioral changes and also increased firing of VTA dopamine neurons [31]. Since BMAL1 and CLOCK are known binding partners presumably they are working together to regulate dopamine levels [32], thus modulate behavioral phenotypes in cKO mice. However, we cannot exclude the potential contribution of other DAT-expressed regions, such as dorsal raphe, to the observed behavioral or neurochemical changes.

Previous works have also shown that dysfunctions in circadian genes are associated with increased dopaminergic functions and related mental disorders. For example, *ClockΔ19* mutant mice exhibit enhanced excitability of dopamine neurons in the VTA and show heightened sensitivity to the rewarding effects of cocaine [33]. *Rev-erbα* suppresses the transcription of TH, the rate-limiting enzyme in dopamine production. Consequently, *Rev-erbα* knockout mice show higher dopamine release and exhibit locomotor hyperactivity and reduced anxiety- and depression-like behaviors [34]. A deficiency in *Per2* leads to elevated dopamine levels, potentially explaining the reduced depression-like behaviors observed in *Per2*-KO mice.

Dopamine homeostasis not only regulates the behavioral phenotypes but also plays a critical role in maintaining the resilience of behavior responsiveness. Our experiments revealed reduced responsiveness to amphetamine in cKO mice, likely due to the already elevated baseline dopamine level before the amphetamine administration. This suggests that the baseline hyperactivity behavior in cKO mice reflects chronic dopamine hypersensitivity, such that amphetamine-induced elevated dopamine level fails to further potentiate dopaminergic responses as it does in control mice. As a result, the dynamic range of dopamine responsiveness is diminished in cKO mice. However, it remains unclear whether similar effects would be observed in female mice, as dopamine release is known to be sexually dimorphic.

Circadian genes also regulate behavioral phenotypes via the modulation of dopamine signaling pathways. Recent studies have shown that *Cry1Δ11* mutants exhibit hyperactive dopamine D1 receptor signaling, resulting in ADHD-like symptoms including hyperactivity, impulsivity and deficits in learning and memory [12]. In our work, we observed hyperexcitability of MSNs in the dorsal striatum, an important downstream target of dopamine neurons. Since the dorsal striatum is a key component of the basal ganglia, which is fundamental to motor control, the hyperactivity of these MSNs in the dorsal striatum likely contributes to the hyperactive behavior observed in cKO mice. Notably, administration of a dopamine D1 receptor antagonist significantly ameliorated hyperactivity in cKO mice, further supporting the critical involvement of D1 receptor signaling in locomotor regulation. However, the same treatment did not change despair-like or cognitive deficits, suggesting that these behavioral abnormalities may involve additional cell types or brain regions that also receiving dopaminergic inputs, such as lateral habenula, hippocampus and cortex. Indeed, due to the lack of electrophysiological distinction between D1- and D2-MSN populations in our recording paradigm, we cannot exclude the possibility that dopamine signaling through other cell types, such as D2-MSNs, may also contribute to the observed behavioral changes. Moreover, since no significant changes were detected in the excitability of pyramidal cells from PFC, it is unlikely that these cells are the primary drivers of the behavioral phenotypes in cKO mice. Further investigation into these neural circuits will be necessary to fully understand the role of circadian genes in neuropsychiatric function.

## Conclusions

In summary, the selective knockout of circadian gene *Bmal1* in dopaminergic neurons lead to hyperactivity, impaired attention and working memory via the disruption of dopaminergic functions in male mice, including the alteration of dopamine catabolism and baseline release. In addition, dopamine D1 receptor signaling in the dorsal striatum contributes significantly to the observed hyperactive behavior. Our work highlights the critical role of circadian genes in maintaining dopaminergic tone and regulating complex behaviors, underscoring the potential therapeutic relevance of targeting circadian genes for treating ADHD-like symptom and other related neuropsychiatric disorders.

## Materials and methods

### Animals

*Bmal1*-flox mice (B6.129S4 (Cg)-Arntl^tm1Weit^/J; stock number 7668) and *Dat*-Cre mice (B6.SJL-Slc6a3^tm1.1(cre)Bkmn^/J; stock number 6660) were obtained from Jackson Laboratory. The genotyping was performed by PCR as previously described [35, 36]. Mice of the same sex were group housed (3–5 animals per cage) under controlled conditions: temperature (20 °C ± 2 °C), relative humidity (50-60%), and a 12-hour light-dark (LD) cycle (lights on at 7:00 AM and lights off at 7:00 PM). Food and water were provided *ad libitum*. All procedures involving the care and use of animals were approved by the Ethics Committee of School of Life Sciences, Central South University of China. All methods were performed in accordance with approved guidelines.

### Immunofluorescent staining

Mice were anesthetized and perfused intracardially with 4% paraformaldehyde. Brains were removed, postfixed and cryoprotected in 25% sucrose overnight. Coronal Sect. (20 μm thick) containing the VTA region were collected and incubated with primary antibodies to BMAL1 (diluted 1:100; abcam, ab3350), MaoA (diluted 1:200; abcam, ab126751), and TH (diluted 1:500; Millipore, AB152) overnight at 4 °C. The slices were then incubated with Alexa Fluor-conjugated labeled secondary Abs in the dark for 1 h. Nuclei were stained with DAPI. Slices were imaged using a confocal microscope (TCS SP8; Leica). The relative intensity was analyzed by ImageJ.

### RNA isolation, reverse transcription and RT-qPCR

Cells or tissues were lysed with TRIzol^®^ reagent according to the manufacturer’s instructions. Two micrograms of total RNA were reversed transcribed using RevertAid First Strand cDNA Synthesis Kit (Thermo Scientific; K1622), and 20 ng of total cDNA equivalents were then analyzed using Fast SYBR™ Green Master Mix (Thermo Scientific; 4385612) according to the manufacturer’s instructions using a C1000 touch Thermal Cycler. Primers used for qPCR were: mPer2 forward 5’-GAAAGCTGTCACCACCATAGAA-3’ and mPer2 reverse 5’-AACTCGCACTTCCTTTTCAGG-3’; mCry1 forward 5’-CACTGGTTCCGAAAGGGACTC-3’ and mCry1 reverse 5’-CTGAAGCAAAAATCGCCACCT-3’; mClock forward 5’- ATGGTGTTTACCGTAAGCTGTAG-3’ and mClock reverse 5’- CTCGCGTTACCAGGAAGCAT-3’. All reactions were performed in triplicate. The relative levels of gene mRNAs were normalized to the corresponding glyceraldehyde3-phosphate dehydrogenase (GAPDH) levels.

### Elisa

Dopamine levels in the nucleus accumbens (NAc) was measured using an enzyme-linked immunosorbent assay (ELISA) kit (Jiangsu Meimian Industrial Co. Ltd., China, MM-0626M1). Tissue specimens were collected, and all steps were performed according to the manufacturer’s instructions. The content of dopamine was detected within 15 min after adding the stop solution, and the absorbance (OD) of each well was measured at 450 nm using an absorption photomicroplate reader (BioTek Synergy 2; Agilent Technologies).

### Circadian behaviors

Male control or cKO (4–5 months) mice were individually housed in cages equipped with running wheels, with free access to food and water. Their locomotor activities were recorded as revolutions per 5-minute intervals. Mice were entrained to an initial LD cycle (light intensity ~ 150 lx, lights on at 7:00 AM and lights off at 7:00 PM). After 2–3 weeks of recording under LD conditions, the mice were transferred to constant darkness (DD) for 3–4 weeks. The free-run period was calculated using ClockLab software (Actimetrics) in the MATLAB environment (MathWorks).

### Behavioral tests

Male control and their littermate cKO mice aged 8–12 weeks were used for behavioral tests. Each mouse (not subjected to drug treatment) was tested in the following order: OFT—Y-maze—TST—Social interaction test. Mice were allowed to rest for 1–2 days between each behavioral test.

#### Open field test (OFT)

Locomotor activity was measured by the OFT. On test day, male control and their littermate cKO mice were transported to the testing room and left undisturbed for 1 h before testing. Each animal was placed in the center of a square arena (72 × 72 × 36 cm), and video recordings for 10 min, 30 min and 90 min respectively. The total distance traveled and number of central zone (36 × 36 cm) crossings were calculated [12].

#### Y-maze test

The Y-maze spontaneous alternation paradigm assesses working memory based on the natural tendency of rodents to explore a novel environment. The apparatus consisted of three opaque arms (30 × 5 × 15 cm) radiating from the center in a Y shape. The behavioral test was initiated by placing the mouse in the center of the Y-maze, allowing free access to all three arms. The movement of the mouse was tracked by a video camera for 8 min and quantified. An arm entry was counted when all 4 paws of the mouse entered an arm, and a “spontaneous alternation” was defined as a sequence of consecutive arms entries without immediate repetition [37]. The number of alternations was determined by counting all valid triplets in the sequence. The total possible alternations were calculated as the total number of arm entries minus 2, as each triplet requires three consecutive entries. The percentage alternation was then computed using the formula: percentage alternation = (Number of Alternations / Total Possible Alternations) × 100.

#### Tail suspension test (TST)

Male control and their littermate cKO mice were individually suspended by their tail in a white box (40 × 20 × 8 cm) for 6 min. Immobility was defined as the cessation of agitation and escape attempts. The time spent immobile during the last 4 min was quantified [12].

#### Reciprocal interaction test

A male mouse was placed in a cage and allowed to habituate for 10 min. Then, a novel conspecific mouse, matched for genotype and age, was introduced into the neutral arena. Social interaction behaviors, including touching, close following, nose-to-anus sniffing, nose-to-nose sniffing, grooming and/or crawling over/under each other were measured. Two independent observers, blinded to the genotypes, quantified the duration of social interactions [38].

#### Bright light stimulation test

As previously described [18, 19], the test was conducted in a white, rectangular, translucent polyethylene box (69 × 34 × 30 cm). Three lamps are placed at the end of table, each containing a white light bulb of 100 W. One lamp was placed 14 cm from the center of a short wall. Two lamps flanked in each long side of the arena respectively, both positioned 14 cm from its long wall and 15 cm from its short wall. Three lamps were mounted on a pole 18 cm above the base of the arena. The test consisted of three continuous phases. Phase 1 and phase 3 is the dark phase (< 0.5 fc) while the lights turn on in the phase 2 (115 fc at one end of the box with light and 85 fc at one end of the box without light). Each phase lasted for 4 min. The change of distance moved was calculated by moved distance per minute in phase 2 minus average distance moved in phase 1. The percentage of change in locomotion was calculated by 100%*(Phase 2 - Phase 1)/Phase 1.

#### Drugs and pharmacological treatments

Mice treated with drugs were tested for only one experiment. SCH23390 (15 µg/kg; Selleck, S0476) and amphetamine (0.2 mg/kg; Changsha Public Security Bureau, China) were dissolved in saline and injected i.p.

### Fiber photometry of dopamine release

To visualize dopamine dynamics in vivo using fiber photometry, AAV9-hSyn-rDA3m (300 nl, titer of 2–6 × 10^12^ vg/ml; BrainCase) was injected into the dorsal striatum (Coordinates relative to bregma in mm: AP + 1.0, ML -2.35, DV -3.3). After the injection, a fiber optic cannula (1.25 mm in diameter, 0.37 NA; ThinkerTech) was implanted 100 μm above the injection site and then secured with dental cement (ChangShu ShangChi Dental Materials). Behavior tests were conducted 3–4 weeks after the surgery. The photometry signal was collected by a multi-channel fiber photometry system (ThinkerTech) via a mono optic fiber. The dopamine sensor rDA3m was excited by a 580-nm laser with a power of ~ 30 µW at the tip (tested during constant light). A top-view recording camera (Huiboshi X20) was used to assess the behavior of animal in an open filed. Mouse behaviors were analyzed frame by frame, and the resting states were defined as periods of immobility lasting longer than 30 s.

For analysis, raw photometry signals from all channels were first processed with polynomial correction using Matlab (2024a, MathWorks) to remove the fluorescent bleaching effect. Next, the corrected signal (from 580 nm channel) was subtracted from the control signal (from 405 nm channel) to obtain ΔF. This value was then divided by the control signal to obtain the change in dopamine release, expressed as ΔF/F_0_. The standard deviation of ΔF/F_0_ traces was estimated as the dopamine release level. The positive standard deviation value was set as a threshold to capture high-level dopamine release bouts. Hyperactive dopamine release was reflected by bigger peaks and longer durations of these release bouts.

### Electrophysiological recording and analysis

Electrophysiological experiments were performed in coronal brain slices containing dorsal striatum. The recording was performed at ZT09-12 timepoint. Brain slices (300–350 μm thick) were prepared from 6- to 8-week-old mice. Mice were deeply anesthetized with sodium pentobarbital (50 mg/kg, i.p.) before decapitation. The brains were rapidly removed and immersed in an ice-cold choline-based artificial cerebrospinal fluid (ACSF) containing 120 mM Choline chloride, 2.4 mM KCl, 7 mM MgCl_2_·6H_2_O, 0.5 mM CaCl_2_, 1.25 mM NaH_2_PO_4_·2H_2_O, 5 mM sodium ascorbate, 3 mM sodium pyruvate, 26 mM NaHCO_3_, 25 mM dextrose and pH 7.2–7.3. Brain blocks were sliced in this ice-cold ACSF using a vibratome (7000 smz-2, Campden Instruments). After slicing, the sections were immediately transferred to an incubation chamber filled with normal ACSF (124 mM NaCl, 2.883 mM KCl, 26 mM NaHCO_3_, 1.25 mM NaH_2_PO_4_·2H_2_O, 10 mM dextrose, 2 mM CaCl_2_, 1.2 mM MgCl_2_·6H_2_O, pH 7.2–7.3). Slices were maintained at 35.5℃ for 45–60 min and then kept at room temperature until use. During the slicing and recording, solutions were continuously aerated with a mixture of 95% O_2_ and 5% CO_2_. Slices were perfused with normal ACSF at 34℃-35℃, at a flow rate of 2 mL/min.

For whole-cell recording, patch pipettes with an impedance of 4–7 MΩ were filled with normal internal solution containing (in mM) 140 K-Gluconate, 3 KCl, 2 MgCl_2_·6H_2_O, 0.2 EGTA, 10 HEPES, and 2 Na_2_ATP (285–295 mOsm, pH 7.2–7.25). Neurons in dorsal striatum were visually identified under a microscope equipped with infrared differential interference contrast (IR-DIC) optics (BX-51WI, Olympus). Recording with series resistance less than 20 MΩ were included for data analysis. Current-clamp recordings were achieved using a MultiClamp 700B amplifier (Molecular Devices). Signals were acquired using Digidata 1550B with pClamp 10.6 software. Signals were sampled at 50 kHz and filtered at 10 kHz.

To examine electrophysiological properties such as the frequency-current (F-I) curve and input resistance, positive (50 pA/step, 500 ms duration) or negative (-50 pA, 500 ms) current pulses were applied. The resting membrane potential (RMP) was defined as the cell membrane potential without any current injection. A successful action potential (AP) was defined as having an amplitude surpassed 30 mV. The rheobase was defined as the minimum current pulse size to initiate an AP. The liquid junction potential (~ 15.6 mV) was not corrected in the data analysis process.

### Statistics

Data analysis was performed using MATLAB (2024a, MathWorks) and Prism 9 (GraphPad Software). All measurements were taken from distinct samples. Shapiro-Wilk test was used to check the normality of the samples. For groups with a normal distribution, two-tailed unpaired t-test was applied; for groups that did not meet normality criteria, the Mann-Whitney U test was used. Statistical significance was set at alpha value = 0.05. Significance levels are denoted as follows: * *P* < 0.05, ** *P* < 0.01, *** *P* < 0.001. Experimental data in the text and figures are presented as mean ± SEM.

## Electronic supplementary material

Below is the link to the electronic supplementary material.

Supplementary Material 1

Supplementary Material 2

## Data Availability

Data is provided within the manuscript or supplementary information files.

## Abbreviations

ADHD: Attention-deficit hyperactivity disorder
Amph: Amphetamine
AP: Action potential
cKO: Conditional knockout
Dat/DAT: Dopamine transporter
DD: Constant darkness
i.p.: Intraperitoneal
LD: Light/dark
MSNs: Medium spiny neurons
OFT: Open field test
PFC: Prefrontal cortex
Rin: Input resistance
RMP: Resting membrane potential
SCH: SCH23390
SNc: Substantia nigra pars compacta
TST: Tail suspension test
VTA: Ventral tegmental area
ZT: Zeitgeber time
