# Supplementary material for: Knockout of Bmal1 in dopaminergic neurons induces ADHD-like symptoms via hyperactive dopamine signaling in male mice
Source: Behav Brain Funct. 2025 Jul 11;21:22. doi: 10.1186/s12993-025-00287-w (PMC12247330; doi:10.1186/s12993-025-00287-w)
Supplement: Supplementary file 2 — Supplementary Material 2 [file 12993_2025_287_MOESM2_ESM.docx]

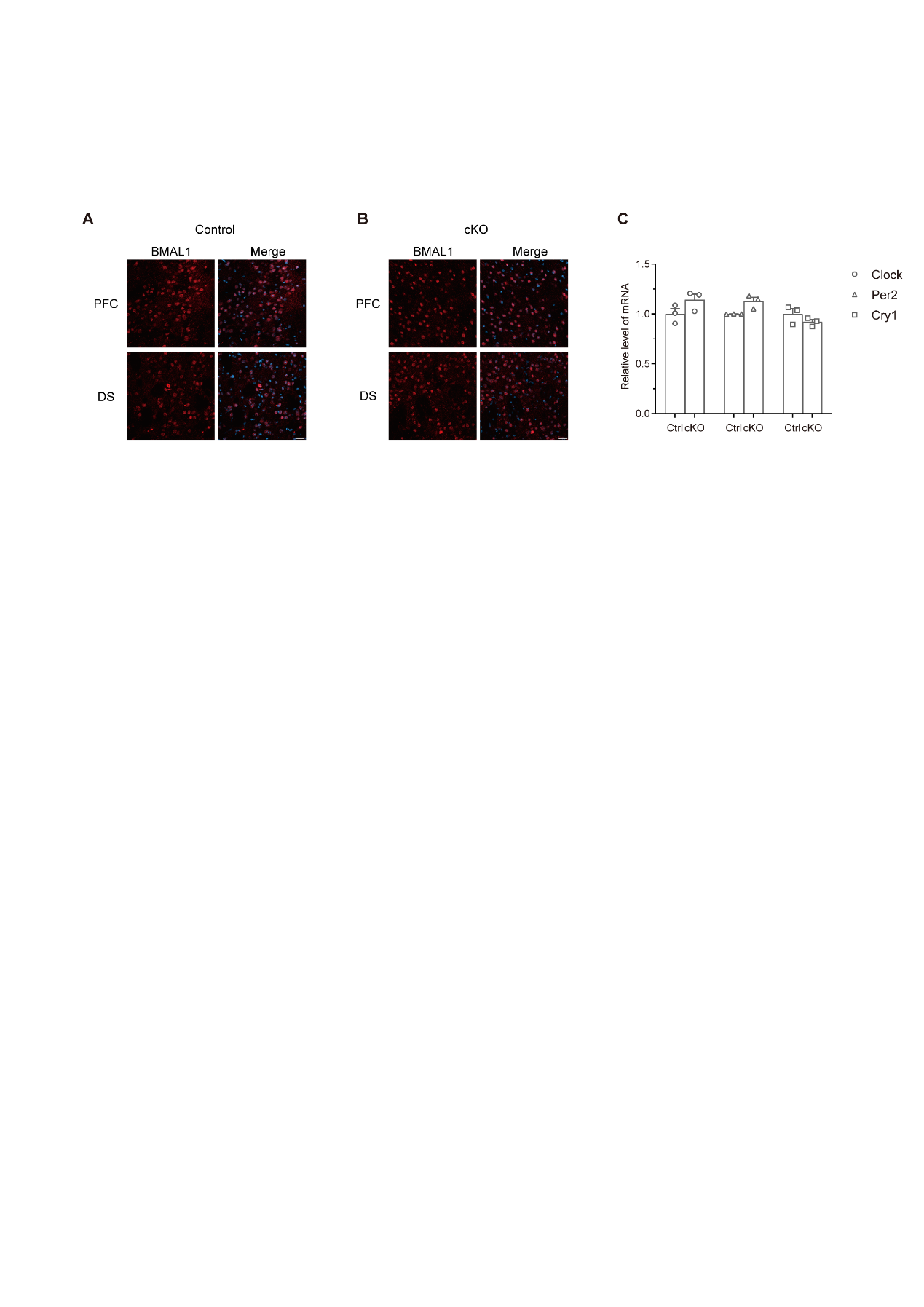
**Supplementary Figure 1. *Bmal1*-cKO does not affect BMAL1 expression in the cortex and dorsal striatum or several circadian gene expressions in midbrain**

(A) Representative immunofluorescence image of BMAL1 expression in prefrontal cortex (PFC) and dorsal striatum (DS) brain regions of Ctrl mice. Red represents BMAL1 protein. Scale bar: 10 μm. (B) Representative immunofluorescence image of BMAL1 expression in PFC and DS brain regions of cKO mice. Red represents BMAL1 protein. Scale bar: 10 μm. (C) Quantitative PCR analysis of the clock genes in the midbrain from control and cKO mice. n = 3 per genotype. Data are presented as mean ± SEM.

**
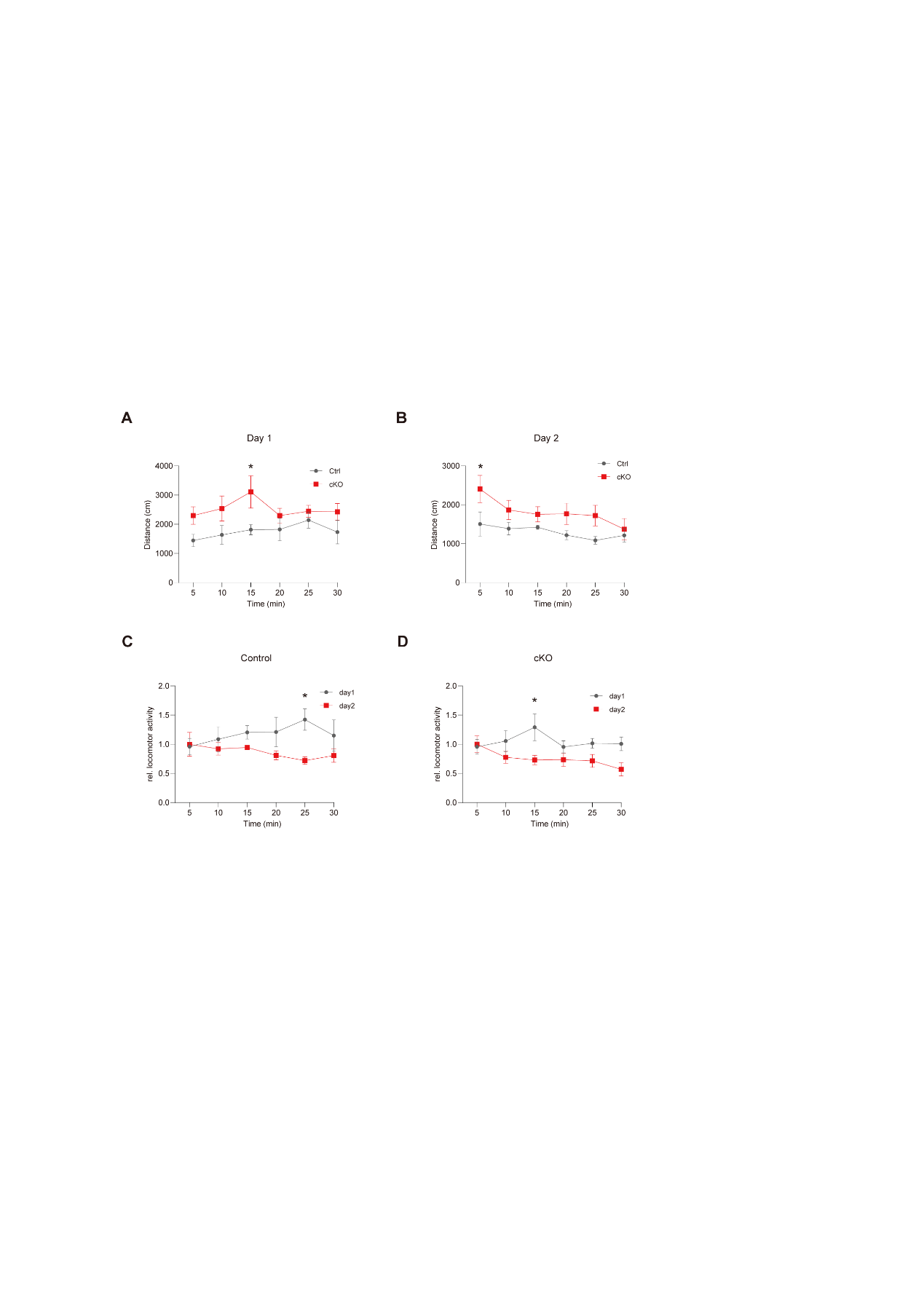
Supplementary Figure 2. *Bmal1*-cKO mice exhibit normal habituation to a novel environment.**

(A) On day 1, cKO mice traveled significantly longer distances than Ctrl mice during a 30-minute OFT; *P < 0.05, repeated-measure ANOVA test. n = 5 mice per genotype. (B) On day 2, cKO mice traveled significantly longer distances than Ctrl mice during a 30-minute OFT; *P < 0.05, repeated-measure ANOVA test. n = 5 mice per genotype. (C) Relative locomotor activity (normalized the distance by setting the first 5-minute distance on day 2’s data to 1) of control mice on day 1 and 2; *P < 0.05, repeated-measure ANOVA test. n = 5 mice per day. (D) Relative locomotor activity (normalized the distance by setting the first 5-minute distance on day 2’s data to 1) of cKO mice on day 1 and 2; *P < 0.05, repeated-measure ANOVA test. n = 5 mice per day. Data are presented as mean ± SEM.


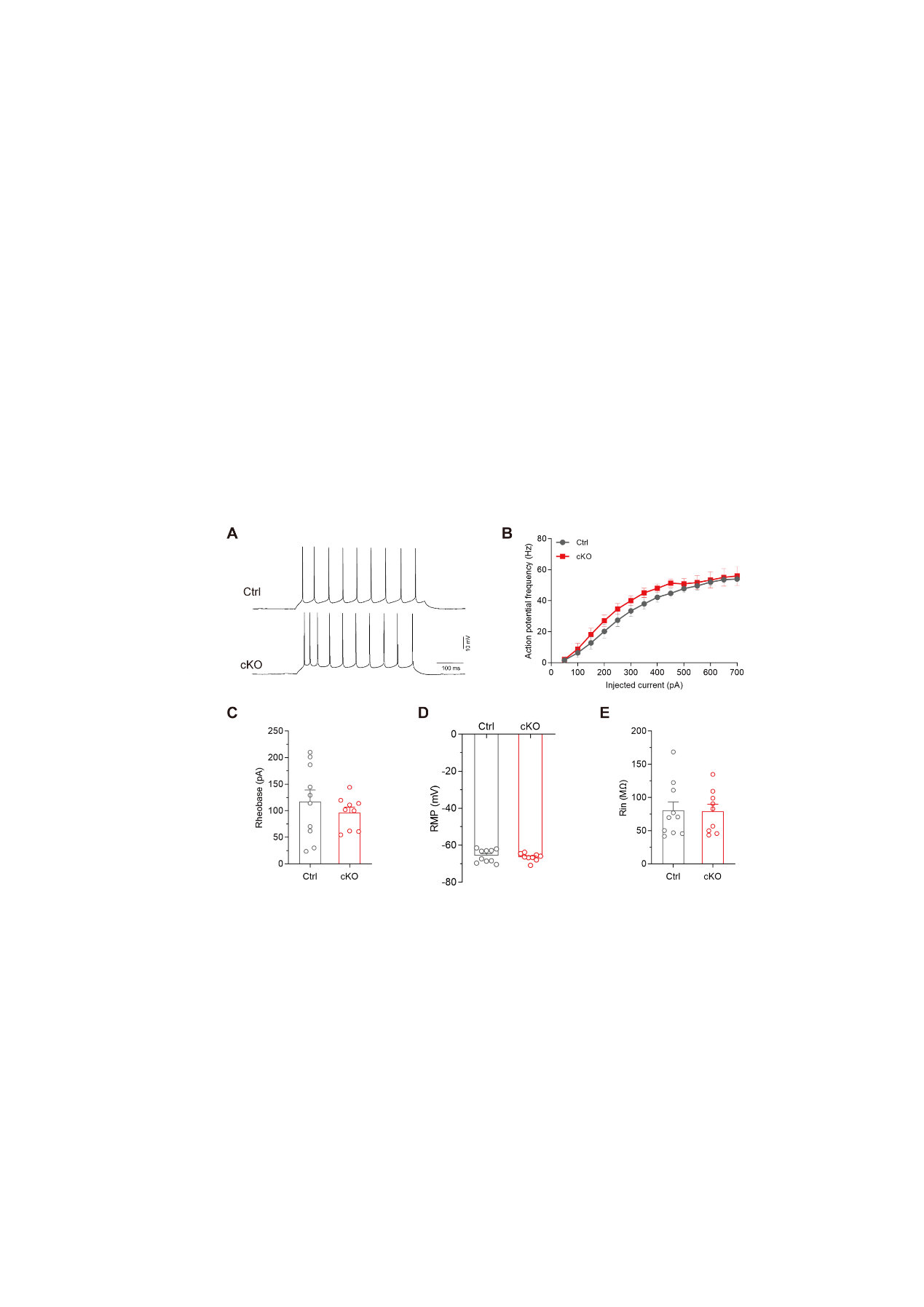
**Supplementary Figure 3. *Bmal1* conditional knockout does not alter the neuronal excitability of pyramidal cells in PFC.**

(A) Representative APs of pyramidal cells in PFC induced by current injections of 100 pA in Ctrl and cKO mice. The recording was performed at ZT09-12 timepoint. (B) The AP frequencies in response to a series of current pulses injections from two groups. n = 10 cells from 2 Ctrl mice versus 9 cells from 2 cKO mice. (C) The rheobase of pyramidal cells from two groups. (D) The resting membrane potential (RMP) of pyramidal cells from two groups. (E) The input resistance (Rin) of pyramidal cells from two groups. Data are presented as mean ± SEM.
